# Partial Dosage Compensation in Strepsiptera, a Sister Group of Beetles

**DOI:** 10.1093/gbe/evv008

**Published:** 2015-01-18

**Authors:** Shivani Mahajan, Doris Bachtrog

**Affiliations:** Department of Integrative Biology, University of California Berkeley

**Keywords:** dosage compensation, sex chromosomes, beetles

## Abstract

Sex chromosomes have evolved independently in many different taxa, and so have mechanisms to compensate for expression differences on sex chromosomes in males and females. Different clades have evolved vastly different ways to achieve dosage compensation, including hypertranscription of the single X in male *Drosophila*, downregulation of both X’s in XX *Caenorhabditis*, or inactivation of one X in female mammals. In the flour beetle *Tribolium*, the X appears hyperexpressed in both sexes, which might represent the first of two steps to evolve dosage compensation along the paths mammals may have taken (i.e., upregulation of X in both sexes, followed by inactivation of one X in females). Here we test for dosage compensation in Strepsiptera, a sister taxon to beetles. We identify sex-linked chromosomes in *Xenos vesparum* based on genomic analysis of males and females, and show that its sex chromosome consists of two chromosomal arms in *Tribolium*: The X chromosome that is shared between *Tribolium* and Strepsiptera, and another chromosome that is autosomal in *Tribolium* and another distantly related Strepsiptera species, but sex-linked in *X. vesparum*. We use RNA-seq (RNA sequencing) to show that dosage compensation along the X of *X. vesparum* is partial and heterogeneous. In particular, genes that are X-linked in both beetles and Strepsiptera appear fully dosage compensated probably through downregulation in both sexes, whereas genes on the more recently added X segment have evolved only partial dosage compensation. In addition, reanalysis of published RNA-seq data suggests that *Tribolium* has evolved dosage compensation, without hypertranscribing the X in females. Our results demonstrate that patterns of dosage compensation are highly variable across sex-determination systems and even within species.

## Introduction

Heteromorphic sex chromosomes have arisen independently in many species from ordinary autosomes (Bull 1983; Charlesworth 1996). Sex chromosome evolution is characterized by a loss of gene function along the nonrecombining Y chromosome (Y degeneration; see Bachtrog [2013] for a recent review). In many organisms with heteromorphic XY sex chromosomes, mechanisms have evolved that equalize expression of X-linked genes in males and females (dosage compensation; Charlesworth 1996; Vicoso and Bachtrog 2009).

Dosage compensation has evolved in response to reduced gene dose of X-linked genes in males, due to loss of Y-linked genes (Charlesworth 1996; Vicoso and Bachtrog 2009). The most direct way to achieve dosage compensation is to simply upregulate X-linked genes in males only, to restore correct levels of X-linked gene product in males, as has evolved in *Drosophila* (Gelbart and Kuroda 2009). However, in the other model systems where dosage compensation has been well studied—mammals and *Caenorhabditis*—the dosage compensation mechanisms operate by reducing expression of the X chromosome in XX females (or hermaphrodites), with mammals completely inactivating one of the two X’s in females, and *Caenorhabditis* halving expression of each X in a hermaphrodite (Meyer 2000; Heard and Disteche 2006).

Halving expression of the X in females presents somewhat of an evolutionary conundrum. If dosage compensation evolved to counterbalance reduced expression of X-linked genes in males in response to Y degeneration and to restore the correct balance between X-linked and autosomal gene products in males, the downregulation of gene expression on the X in females does not solve the gene dose problem that males experience. Instead, it simply creates the same gene dose deficiency and X-autosome imbalances of gene products in females. It has thus been proposed that dosage compensation in mammals and *Caenorhabditis* evolved in a two-step process (Ohno 1967; Charlesworth 1996; Vicoso and Bachtrog 2009; Mank et al. 2011; Mank 2013). In response to Y degeneration, the X first became upregulated in both sexes. This would have resolved the gene dose deficiency that is experienced by males, but would also result in too much gene product in females. In response to overexpression in females, X downregulation or X inactivation has evolved secondarily, to restore correct X-autosome gene balance in females (Ohno 1967; Charlesworth 1996; Vicoso and Bachtrog 2009).

In both mammals and *Caenorhabditis*, only the second step of dosage compensation is well understood, and there has been considerable debate on whether there is global upregulation of X-linked genes relative to its ancestral expression level (Nguyen and Disteche 2006). Depending on the data set used, and statistical analysis of sometimes the same data, different studies have yielded opposite conclusions as to whether mammals globally upregulate their X chromosome in both sexes, relative to its ancestral expression level, or relative to autosomal expression, or whether only a subset of dosage-sensitive genes are upregulated on the X (Pessia et al. 2012). Most recent studies have concluded that placental mammals do not globally upregulate their X chromosome, but instead, only a subset of X-linked genes that are part of protein-complexes appear to be upregulated in both sexes (Lin et al. 2012; Pessia et al. 2012), whereas a subset of autosomal genes interacting with X-linked genes were found to have become downregulated in placentals upon the emergence of sex chromosomes (Julien et al. 2012). Marsupials, on the other hand, may globally upregulate their X in both sexes, relative to ancestral expression levels (Julien et al. 2012). Thus, the mechanisms of dosage compensation, and the evolution of X inactivation in mammals remain controversial (Julien et al. 2012; Pessia et al. 2013). Studying additional taxa with independently formed sex chromosomes should help to identify general principles driving the evolution of dosage compensation.

Karyotypes in Coleoptera have been well-studied, and almost all beetles have heteromorphic sex chromosomes (either XX/XY or XX/X0 systems). A recent study in the flour beetle *Tribolium castaneum*, a model species whose genome has been sequenced and annotated, has concluded that dosage compensation in this species evolved by upregulating the X chromosome in a nonsex-specific manner, that is, expression of the X was found to be increased in both males and females (Prince et al. 2010). Although this restores correct expression of X-linked genes in males, it also leads to hypertranscription of the X in females (Prince et al. 2010), and may thus represent the first of two steps to evolve dosage compensation along the paths marsupials may have taken. Here we identify sex-linked genes and analyze male and female gene expression in twisted-wing insects (Strepsiptera), a sister taxon to beetles (Coleoptera), to better understand the evolutionary forces driving dosage compensation in this group.

Strepsiptera are a morphologically highly derived group of endoparasitic insects whose phylogenetic position was debated for some time, but the most recent and complete studies clearly support a sister relationship of Strepsiptera with Coleoptera (Niehuis et al. 2012; Boussau et al. 2014). Strepsiptera have separate sexes, and cytogenetic data for this group exist for just two species but indicate the presence of heteromorphic X and Y chromosomes. In particular, the diploid chromosome number of *Xenos peckii* was identified as 16, and in an unidentified species of *Xenos* from Brazil, three pairs of autosomes and an XY sex chromosome were reported (Ferreira et al. 1984). Here we use genomic sequencing of the Strepsiptera *Xenos vesparum* (family Stylopidae), and we also analyze published genome data from *Mengenilla moldrzyki* (Niehuis et al. 2012), a species belonging to the early-divergent Strepsipteran family Mengenillidae, to identify the sex chromosomes of Strepsiptera, and gene expression analysis in *X. vesparum* and *T. castaneum* to investigate the absence or presence of dosage compensation.

## Materials and Methods

### Sampling and Sequencing of Strepsiptera

We sequenced the DNA from an adult male (library insert size 700–800 bp) and two females of *X. vesparum* (neotenic adult female with library insert size 700–800 bp and female fourth instar larva with library insert size of 250 bp). For gene expression analysis, we prepared libraries for two female samples (neotenic adults, and fourth instar larvae; library insert size about 200 bp), and one male sample (pupae). DNA was extracted using Puregene, with proteinase K and RNAse A treatment during lysis, and was purified with overnight Isopropanol precipitation. RNA was extracted with Trizol, and purified overnight with Ethanol precipitation. For both the DNA and RNA extraction, purity measurement and quantification was done using Nanodrop and Qubit. The Libraries were prepared using standard Illumina TruSeq kits and protocols, and the cleanup was done using AmpureXP, followed by size-selection of the DNA libraries on agarose gels. We obtained 27,578,418 genomic reads for the adult female; 77,729,238 reads for male; and 19,045,611 reads for female larva. After RNA sequencing (RNA-seq) we obtained 100,160,332 reads for the neotenic adult female; 314,698,728 reads for male; and 283,804,476 reads for female fourth instar larva. The genome assembly of *M. moldrzyki* was obtained from http://datadryad.org/resource/doi:10.5061/dryad.ts058.2 (last accessed February 3, 2015), and unpaired shotgun 454 reads (a total of 5,449,680 reads) from male *M. moldrzyki* samples were provided to us by the authors (Niehuis et al. 2012).

### Genome Assembly and Coverage Analysis to Infer Sex-Linkage

Paired-end reads from the female *X. vesparum* sample were trimmed and assembled using SOAPdenovo (Li et al. 2009) with a K-mer size of 63. Gapcloser was used to further improve the quality of the assembly. The assembled genome contained 11,895 scaffolds and was 81.4 Mb long (supplementary table S1, Supplementary Material online). Only scaffolds greater than 1,000-bp were retained for further analysis. Coding sequences (CDS) from *Tribolium* were used to assign the scaffolds to chromosomes. *Tribolium* CDS were downloaded from ftp://ftp.bioinformatics.ksu.edu/pub/BeetleBase/ (version 3) and mapped to the *X. vesparum* genome using blat with a translated database and query and only the best hit was kept. A scaffold was assigned to the consensus *Tribolium* chromosome in case more than one gene mapped to that scaffold; when only one gene mapped to a scaffold, it was assigned to the chromosome on which that gene was located. A total of 2,291 scaffolds mapped to *T. castaneum* chromosomes and only these were retained for further analysis. Male and female *X. vesparum* trimmed paired-end genomic reads were aligned separately to the de novo assembled *X. vesparum* genome using bwa (Li and Durbin 2009). Scaffold coverage was calculated using soapcoverage. The log (base 2) of the coverage per chromosome was then plotted in R. 454 reads from male *M*. *moldrzyki* samples were aligned to the published reference *M. moldrzyki* genome (Niehuis et al. 2012) using bwa-sw and coverage was calculated using soapcoverage. Sex-linkage of scaffolds was inferred in the same way as for *X. vesparum* using *T. castaneum* CDS. Coverage was normalized by the median scaffold length as well as the median coverage of the autosomes, and log (base 2) of the normalized male coverage was plotted in R.

### Transcriptome Assembly and Gene Expression Analysis

FastQC was used for the quality control of the paired-end reads from the two female (fourth instar larva and neotenic adult) samples and male (adult) sample. The reads were then trimmed, pooled, and assembled using SOAPdenovotrans with a kmer size of 75 (supplementary table S2, Supplementary Material online). The obtained transcripts were mapped to *Tribolium* CDS using Blat with a translated query and database. The Blat output was then filtered and only the best match per transcript was retained. For transcripts overlapping a *Tribolium* gene by more than 20 bp, only the transcript with the highest alignment score was retained. For those that overlapped by less than 20 bp, their sequences were concatenated. Transcripts mapping to different parts of the same gene were also concatenated. Finally, transcripts were assigned the location of their corresponding *Tribolium* genes on the *Tribolium* genome. This resulted in a total of 4,413 genes for *X. vesparum*. Trimmed male and female paired-end RNA-seq reads were aligned to the de novo assembled transcriptome using bowtie2 (Langmead and Salzberg 2012) and FPKM values were calculated using eXpress (Roberts and Pachter 2013). The log (base 2) of the FPKM values per chromosome was then plotted in R. We also analyzed published RNA-seq reads from *T. castaneum* (Li et al. 2013). Unpaired RNA-seq reads from male and female abdominal and prothoracic glands were downloaded from National Center for Biotechnology Information (NCBI) SRA (http://www.ncbi.nlm.nih.gov/sra/, last accessed February 3, 2015; accession numbers: SRX501821, SRX501822, SRX501819, and SRX501820). For each gland, male and female reads were separately mapped to *Tribolium* CDS using bowtie2 and FPKM values were calculated using the eXpress package and plotted in R.

## Results

### Identification of Sex Chromosomes in Strepsiptera

**Fig. 1.—:**
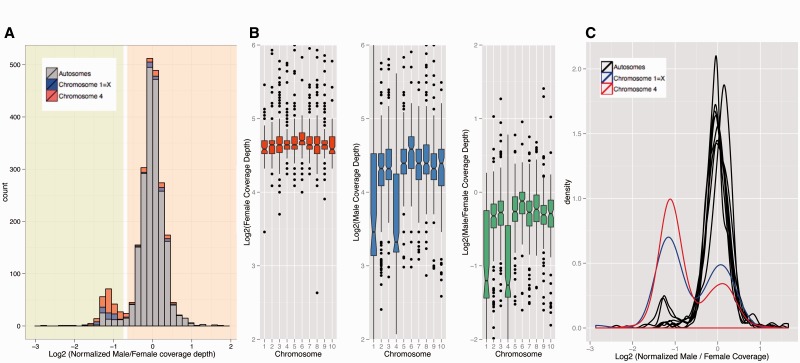
Male and female genomic coverage analyses to identify sex chromosomes in *Xenos*. (*A*) Histogram of Log2 male/female coverage. Scaffolds that map to chromosomes 1 and 4 of *T. castaneum* are shown in blue and red, respectively. The bimodal distribution in coverage suggests that a substantial fraction of the genome is sex-linked in *Xenos*, with the peak with reduced male/female coverage corresponding to scaffolds that are X-linked in *X. vesparum*. (*B*) Boxplot of log2 of coverage in female (in red), male (in blue), and male/female (in green). Overall, there is a drop in male/female coverage for scaffolds that map to chromosomes 1 and 4 in *Tribolium*, suggesting that these chromosomal elements are X-linked in *X. vesparum*. (*C*) Density plot of log2 normalized male/female coverage. Normalization was done by dividing the coverage of scaffolds in each chromosome by the median of the coverage of all scaffolds in chromosomes other than chromosome 1 and chromosome 4. Distributions of chromosome 1 and chromosome 4 are different from that of other chromosomes. The bimodal shape suggests that there have been some rearrangements in the *X. vesparum* genome compared with the genome of *T. castaneum*.

To infer sex chromosomes in *Xenos*, we used genomic read coverage in males versus females (Vicoso and Bachtrog 2013). In particular, regions that are autosomal should show equal read coverage in both sexes, whereas X-linked regions only have half the coverage in males relative to females. Indeed, we find a bimodal distribution of male/female coverage of scaffolds, indicating that a substantial fraction of the genomic scaffolds are X-linked in *Xenos* (fig. 1*A*). To order scaffolds from Strepsiptera, we mapped them against chromosomes from *Tribolium*. *Tribolium castaneum* contains ten similarly sized chromosome pairs, one of which (chromosome 1) segregates as the X chromosome. We find that scaffolds mapping to two chromosome elements from *Tribolium* show reduced male/female read coverage in *X. vesparum*, suggesting that the sex chromosomes of *Xenos* correspond to two different chromosomes of *Tribolium* (fig. 1*B* and *C*; supplementary fig. S1, Supplementary Material online). One of the X-linked elements of *X. vesparum* is also the X chromosome of *Tribolium*, which suggests that this chromosome may already have been a sex chromosome in an ancestor of beetles and Strepsiptera, and thus may have been segregating as a sex chromosome for over 250 Myr (Wiegmann et al. 2009). The other sex-linked element of *X. vesparum* corresponds to chromosome 4 of *Tribolium*, suggesting that this element became X-linked only after the split of Coleoptera and Strepsiptera. Coverage analysis of genomic reads from male *M. moldrzyki* (Niehuis et al. 2012), a species belonging to the early-divergent Strepsipteran family Mengenillidae, shows that only scaffolds that map to chromosome 1 of *Tribolium* have reduced read coverage in male *M. moldrzyki* (supplementary fig. S2, Supplementary Material online). This supports that chromosome 1 is an ancient sex chromosome in Strepsiptera, and also shows that chromosome 4 only became X-linked in an ancestor of *Xenos* after the divergence of the two families Mengenilidae and Stylopidae about 50 Ma (Wiegmann et al. 2009). We refer to these segments as the ancestral region (homologous to chromosome 1 of *Tribolium*) and more recently added region (homologous to chromosome 4 of *Tribolium*) of the X chromosome of *Xenos*.

**Fig. 2.—:**
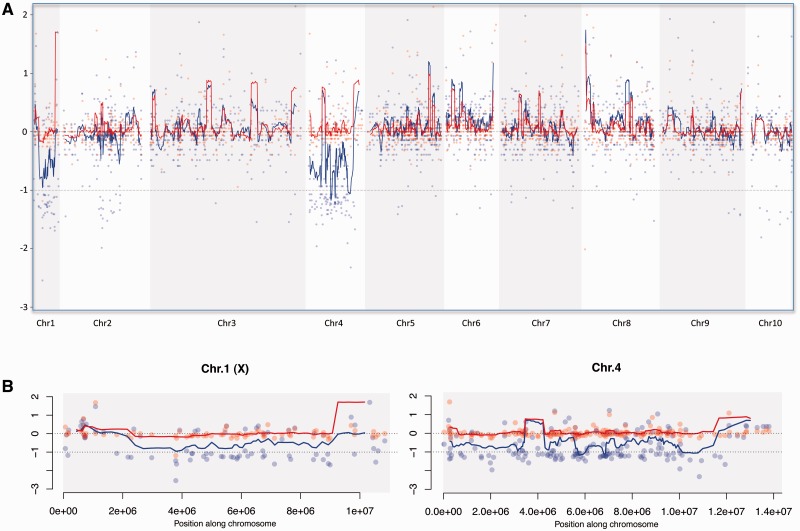
Sliding window analysis of scaffolds mapped along the *Tribolium* genome. (*A*) Log2 of coverage densities of males (in blue) and females (in red) for the scaffolds that mapped to one of the ten chromosomes in *T. castaneum*. The lines represent a sliding window along the chromosomes, with a window size of ten genes. Chromosomes 1 and 4 show a clear drop in coverage for males as compared with females. (*B*) Same as (*A*) but zoomed in into chromosome 1 and chromosome 4.

To investigate whether segments of other chromosomes also show reduced coverage, and whether coverage along chromosomes 1 and 4 is reduced uniformly, we mapped our *Xenos* scaffolds along the *Tribolium* genome (fig. 2; supplementary figs. S3 and S4, Supplementary Material online). In general, we find no evidence of large genomic segments from other chromosomes to show reduced coverage in males versus females (fig. 2*A*). Thus, although it is certainly the case that individual genes from these other chromosomes are also sex-linked in *Xenos*, most of them appear to be indeed autosomal in *X. vesparum.* On the other hand, coverage along chromosomes 1 and 4 is reduced relatively uniformly (fig. 2*B*), suggesting that most genes located on these chromosomes are sex-linked in *Xenos*. Nevertheless, some scaffolds on chromosomes 1 and 4 clearly show coverage levels that suggest that they are autosomal in *Xenos* (fig. 1*C*), indicating that some genomic rearrangements have taken place between these species. We therefore used two approaches to identify X-linked and autosomal genes in *Xenos*: 1) Genes were considered X-linked if their reciprocal-best-hit in *Tribolium* was located on chromosome 1 or chromosome 4; and 2) genes were classified as X-linked if they were located on a scaffold that had reduced male/female coverage, as shown on figure 1*A*: Scaffolds in the green shaded area were classified as X-linked, scaffolds in the orange shaded area were classified as autosomal. Both classifications were used to compare the male and female expression of genes on the X and autosomes of *Xenos*, and both yielded similar results (see below).

### Gene Expression Analysis in Strepsiptera and *Tribolium*

**Fig. 3.—:**
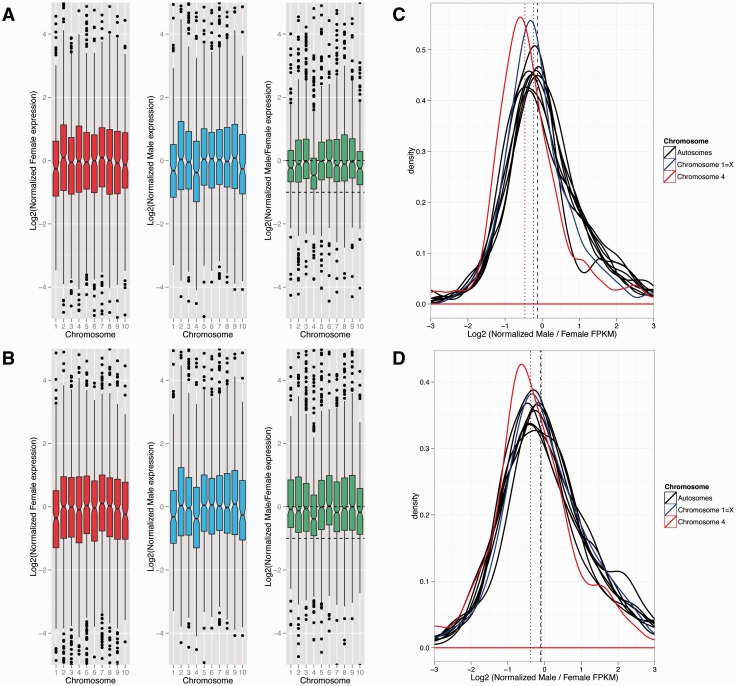
Dosage compensation analysis. Boxplot of log2 of expression in (*A*) fourth instar female larva and (*B*) neotenic adult female (in red), male (in blue), and male/female (in green). FPKM cutoffs for each sample were determined based on the FPKM values for introns and intergenic regions (see supplementary fig. S5, Supplementary Material online). The distribution of log2(male/female) FPKM values for chromosome 4 is significantly different than that of the autosomes with a Wilcoxon test (*P* value of < 2.2e-16 for fourth instar female larva; *P* value of 4.297e-13 for neotenic adult female). There was no significant difference observed for any other chromosome in either comparison (corrected for multiple testing). This result holds true for several FPKM cutoffs: 0, 1, 10 (see supplementary figs. S6 and S7, Supplementary Material online). Density plot of log2 of (*C*) normalized male/fourth instar female larva FPKM and (*D*) normalized male/neotenic adult female FPKM. Normalization was done by dividing each chromosome by the median of the expression of all genes in chromosomes other than chromosome 1 and chromosome 4. Chromosome 1 is almost completely dosage compensated, whereas chromosome 4 is only partially compensated.

**Fig. 4.—:**
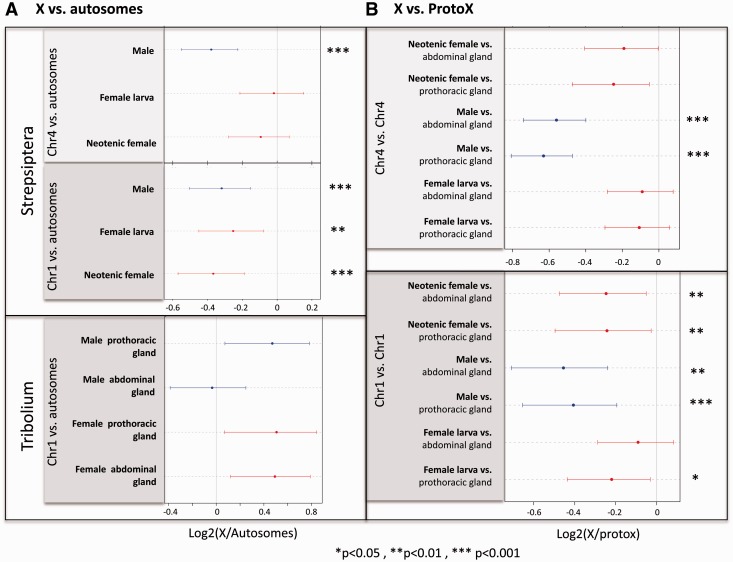
Current and inferred ancestral expression levels of beetle and Strepsiptera sex chromosomes. (*A*) X to autosome expression level ratio of expressed genes on the current sex chromosomes in *Tribolium* and *Xenos*. (*B*) Ancestral expression analysis of *Xenos* sex chromosomes, using expression values in male and female *T. castaneum* as a proxy for ancestral expression values. Expression of genes mapping to chromosome 4 relative to the proto X, and expression of chromosome 1 (which is the ancient sex chromosome) in *X. vesparum* relative to chromosome 1 in *T. castaneum*. Female *X. vesparum* is shown in red and male *X. vesparum* is shown in blue. Expression of each *X. vesparum* sample is compared with the expression in both abdominal (top) and prothoracic glands (bottom) of *Tribolium* for each sex separately. Asterisks are used to denote cases where a significant increase or decrease in expression is observed relative to the ancestral expression (*P < 0.05, **P < 0.01, ***P < 0.001). Dots represent the median, and bars their approximate confidence interval (median ± 1.57 × IQR/√n, where IQR is the interquantile range and *n* the sample size; this is equivalent to the notch size of a boxplot).

To assay whether *Xenos* has evolved dosage compensation, we measured gene expression in males and females. FPKM cutoffs for each sample were determined based on FPKM values for introns and intergenic regions (see supplementary fig. S5, Supplementary Material online; note that the results are insensitive to different FPKM cutoffs, supplementary figs. S6 and S7, Supplementary Material online). We assayed gene expression in neotenic adult females, fourth instar female larvae, and male pupae of *X. vesparum* (fig. 3). Expression levels are similar across autosomes in both sexes, and similar to expression levels of genes mapping to *Tribolium* chromosome 4 (the more recently formed X) in females, but reduced in males (Wilcoxon test *P* value 1.2e-07 when comparing expression of chromosome 4 in males vs. autosomes; and *P* values 3.0e-07 and <2.2e-16 when comparing male/neotenic adult female and male/larval female FPKM ratios, respectively, for chromosome 4 vs. the autosomes; fig. 4*A*). Expression from genes mapping to chromosome 1 of *Tribolium* (the ancestral X) is slightly reduced in both sexes, to a similar extent, relative to autosomes (Wilcoxon test *P* values 0.0001, 0.0002 and 0.0256 for male, neotenic adult female and female larva, respectively; fig. 4*A*). Male/female expression ratios for both adult and larval sample are similar across autosomes, and X-linked genes mapping to chromosome 1 of *Tribolium*. Thus, genes on the presumably ancestral X of Strepsiptera and beetles are dosage compensated. In contrast, *X. vesparum* genes mapping to *Tribolium* chromosome 4 show significantly lower male/female expression ratios, using both the adult and larvae female sample (fig. 3). The decrease in expression in males is less than 0.5, suggesting that this more recently formed X chromosome has evolved partial but incomplete dosage compensation. Thus, we find nearly complete dosage compensation on the ancestral X, which is expressed at a lower level in both sexes relative to autosomes, and partial dosage compensation on the more recently added X, which shows lower expression relative to autosomes in males only.

We used expression in *Tribolium* as a proxy to infer ancestral expression levels in *Xenos*. A previous analysis of whole-body adult microarray data showed that *Tribolium* males have similar levels of gene expression at X-linked and autosomal genes, whereas the X appears hypertranscribed in females (Prince et al. 2010). We analyzed published RNA-seq data from prothoracic glands and abdominal glands from male and female *T. castaneum* and, surprisingly, found that expression of genes from the X and the autosomes is similar in both males and females, for both tissues (fig. 4*A*; supplementary figs. S8 and S9, Supplementary Material online). This would suggest that *Tribolium*, at least in its prothoracic and abdominal glands, has evolved dosage compensation without hypertranscribing the X chromosome in females. To test whether dosage compensation in *Xenos* evolved through downregulation of the X in both sexes (as has been suggested in mammals), or through upregulation of the single X in males (as done in *Drosophila*), we used expression from *Tribolium* as a proxy for proto-X expression before the X became sex-linked. Contrasting expression levels of the X on *Xenos* to that of *Tribolium* suggests that genes mapping to chromosome 1 have become downregulated in both male and female *Xenos*, whereas genes mapping to chromosome 4 are expressed at a lower level only in male *Xenos* (fig. 4*B*; supplementary fig. S10, Supplementary Material online). Thus, this supports our conclusion that the ancestral X chromosome of *Xenos* is dosage compensated through downregulation in both sexes, and only partial dosage compensation has evolved on the more recently formed X chromosome. Note that this analysis assumes that gene expression levels on chromosome 1 in *Tribolium*, which is X-linked in both species, reflect ancestral expression levels. As stated above, expression from the X is similar in both male and female *Tribolium*, and similar to autosomes. If expression on the X in females reflects ancestral expression levels in *Tribolium* and dosage compensation simply evolved by upregulating the X in males (as done in *Drosophila*), this would validate the use of *Tribolium* expression data from chromosome 1 as a proxy for ancestral expression levels. It is also possible that expression on chromosome 1 was higher ancestrally, and dosage compensation in *Tribolium* evolved by downregulating the X in both sexes (as has happened in mammals); this would imply that we underestimate the magnitude of downregulation on the *Xenos* X chromosome. However, we cannot exclude the formal possibility that chromosome 1 was expressed at a lower level in an ancestor of beetles and became upregulated in both male and female *Tribolium* after it became a sex chromosome but more so in males (to compensate for gene dose differences between sexes). In this case, expression from chromosome 1 may not have changed in female *Xenos*. Genes on chromosome 4 are autosomal in *Tribolium* and thus should reflect the ancestral proto-X expression level.

**Fig. 5.—:**
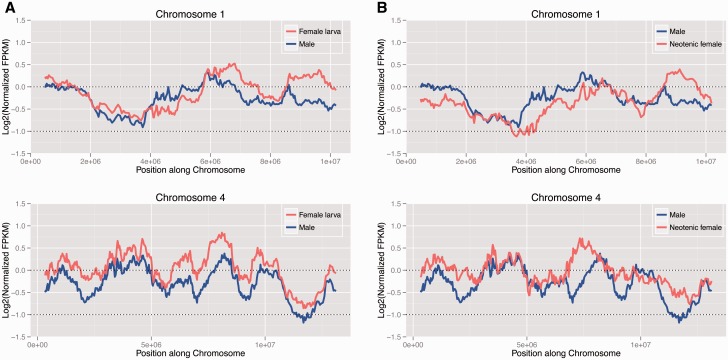
Sliding window analysis of dosage compensation. Log2 of normalized FPKM values of *Xenos* males and females mapped along chromosome 1 and chromosome 4 of *T. castaneum*. Male is shown in blue and (*A*) female larva and (*B*) neotenic adult female are shown in red. Chromosome 1 appears to be almost completely dosage compensated, with some regions showing male-biased and others showing female-biased expression. Chromosome 4, on the other hand, has globally lower FPKM values for males than females, suggesting that complete dosage compensation has not yet evolved on the this chromosome.

To investigate whether dosage compensation is heterogeneous across the X chromosomes with some segments being compensated, we mapped male and female expression levels along the *Tribolium* genome (fig. 5). We find that expression of genes mapping along chromosome 4 is generally female-biased, both in neotenic adult females, and fourth instar female larvae. This is consistent with a global lack of chromosome-wide dosage compensation along this more recently formed X. On the other hand, there is more heterogeneity in expression levels of genes mapping across chromosome 1, with some regions showing male-biased expression, and others showing female-biased expression, resulting in global patterns of dosage compensation on this chromosomal element (fig. 4*B*). This is similar to patterns of sex-biased gene expression seen on autosomes (supplementary figs. S11 and S12, Supplementary Material online).

We repeated the gene expression analysis using only scaffolds that show reduced genomic coverage in males relative to females, and found similar results (supplementary fig. S13, Supplementary Material online). X-linked genes are generally underexpressed in males relative to autosomes, both for genes mapping to the ancestral and the more recently formed X, whereas in females, only genes on the ancestral X are downregulated, relative to autosomes. Thus, the X of Strepsiptera shows partial dosage compensation, and genes mapping to chromosome 1 are compensated more fully than those mapping to chromosome 4. However, dosage compensation on the ancestral X seems less complete using this classification scheme, possibly due to inclusion of some autosomal genes with equal expression in males and females using the first classification method.

## Discussion

Both beetles and Strepsiptera have heteromorphic sex chromosomes, and we show that the X chromosome of *X. vesparum* consists of two chromosomal elements in *Tribolium*. Part of the X chromosome of *X. vesparum* is homologous to the X of *Tribolium*, suggesting that this element was already an X chromosome in the ancestor of beetles and Strepsiptera, and thus has been segregating as a sex chromosome since before these groups split over 250 Ma. A second chromosome that is X-linked in *X. vesparum* is autosomal in both *Tribolium* and in a Strepsiptera species belonging to the early-divergent Strepsipteran family Mengenillidae, which implies that this chromosome became sex-linked more recently, after the split of Mengenilidae and Stylopidae over 50 Ma. Without sampling of additional species, we cannot determine how long ago this second chromosome became incorporated into the X of *X. vesparum*. In species where chromosomes only became sex-linked relatively recently (i.e., in the past 1 Myr), such as the neo-sex chromosomes of several *Drosophila* species, the X and the Y chromosomes still harbor sufficient homology so that some sequencing reads that are derived from the Y chromosome also map to the X chromosome, and genomic coverage is only somewhat reduced for these recently formed neo-sex chromosomes (Zhou and Bachtrog 2012; Vicoso and Bachtrog 2013). Coverage of the more recently formed sex chromosome of *Xenos* is similar to that of the X shared with beetles, which suggests that this segment became X-linked long enough ago for its former homolog (the Y chromosome) to degenerate completely.

Whole-body microarray data suggested that dosage compensation in *Tribolium* involves the upregulation of the X in both sexes (Prince et al. 2010), resulting in female-biased expression of the X. Our analysis of RNA-seq data from prothoracic and abdominal glands, however, indicates that the X and autosomes are transcribed at similar levels in both sexes, that is, dosage compensation has evolved without hypertranscribing the X chromosome in females. It is possible that the different findings are due to differences in methodology, statistical analysis, or the mechanism of dosage compensation or sex-biased expression patterns among tissues. In particular, gonads of many organisms often show an excess of genes with sex-biased expression; *Drosophila* species, for example, often harbor an excess of genes with ovary-biased expression on their X chromosomes (Assis et al. 2012), which could contribute to an excess of X-linked expression in whole-body adult females. On the other hand, testis may lack dosage compensation, as found in *Drosophila* (Rastelli and Kuroda 1998), reducing X-linked expression in whole body adult males. It will be of great interest to study gene expression in additional tissues of *Tribolium* as well as *Xenos*, to establish the mechanisms of dosage compensation and sex-biased expression patterns in these species, and how they vary across tissues.

We find that the two X-linked arms of *Xenos* show different levels of dosage compensation; the X shared between *Tribolium* and Strepsiptera appears to be expressed at a lower level in both male and female *X. vesparum*. Given that RNA-seq data suggest that the X chromosome is expressed at similar levels in male and female *Tribolium*, this probably does not reflect lower ancestral expression of that chromosomal arm, but instead suggests that genes mapping to chromosome 1 became downregulated in both sexes of *Xenos*. Note that we do not have a suitable outgroup species where chromosome 1 is autosomal, so we cannot formally exclude the possibility that this chromosome was expressed at a lower level in an ancestor of beetles and Strepsiptera. Downregulation of the X in females alone, however, does not restore gene dose imbalances between X-linked and autosomal genes, and dosage compensation of the ancestral X in Strepsiptera might involve the downregulation of autosomal genes that interact with genes on the X, and evolve along the following path: Y degeneration creates gene dose imbalances for some networks that utilize both X-linked and autosomal genes in males, and downregulation of autosomal genes that interact with X-linked genes would restore proper X-autosome expression ratios in males. If downregulation of autosomal genes is not sex-specific, this would result in gene dose imbalances for these networks in females, and create selective pressure to downregulate X-linked genes interacting with those autosomal genes in females. The outcome of this evolutionary process would be an X chromosome that is expressed at a lower level in both sexes relative to its ancestral expression level, and the simultaneous downregulation of autosomal genes interacting with X-linked genes, in both sexes.

This path resembles the different proposed mechanisms of dosage compensation of the partially homologous X chromosomes shared by placental mammals and marsupials (Julien et al. 2012; Pessia et al. 2012). Gene expression analyses suggest that the X has become globally upregulated in marsupials followed by X inactivation (Julien et al. 2012), whereas no global upregulation of the X chromosome was found in placental mammals. Instead, a subset of autosomal genes interacting with X-linked genes have become downregulated (Julien et al. 2012), and a subset of X-linked genes that are part of protein-complexes appear to have become upregulated in placentals in both sexes upon the emergence of sex chromosomes (Lin et al. 2012; Pessia et al. 2012). Thus, different solutions were found to equilibrate X expression levels between the sexes in these two lineages, similar to what we find in Strepsiptera and Coleoptera.

Yet, although dosage compensation has evolved on the chromosomal arm that is also X-linked in *Tribolium*, dosage compensation appears incomplete at genes that locate to the more recently added part of the *X. vesparum* X chromosome. Several species with female heterogametic sex determination, including birds (Ellegren et al. 2007), some butterflies (Harrison et al. 2012) and snakes (Vicoso et al. 2013), but also male heterogametic monotremes (Julien et al. 2012), have not evolved chromosome-wide mechanisms to equalize X-linked expression levels in males and females. Incomplete dosage compensation implies that many sex-linked genes have different expression levels in males and females, and gene networks employing X-linked and autosomal genes will differ between sexes (Mank 2013). It is possible that there simply has not been enough time yet for this more recently formed X chromosome to evolve full dosage compensation, as has been proposed for the recently formed X chromosome of threespine sticklebacks (Leder et al. 2010). It will be of great interest to study gene expression patterns in additional species of Coleoptera and Strepsiptera, to identify the strikingly different ways in which dosage alterations associated with the emergence of sex chromosomes were resolved.

## Supplementary Material

Supplementary figures S1–S13 and tables S1 and S2 are available at *Genome Biology and Evolution* online (http://www.gbe.oxfordjournals.org/).

Supplementary Data

## References

[evv008-B1] Assis R, Zhou Q, Bachtrog D (2012). Sex-biased transcriptome evolution in *Drosophila*. Genome Biol Evol..

[evv008-B2] Bachtrog D (2013). Y-chromosome evolution: emerging insights into processes of Y-chromosome degeneration. Nat Rev Genet..

[evv008-B3] Boussau B (2014). Strepsiptera, phylogenomics and the long branch attraction problem. PLoS ONE.

[evv008-B4] Bull JJ (1983). Evolution of sex determining mechanisms.

[evv008-B5] Charlesworth B (1996). The evolution of chromosomal sex determination and dosage compensation. Curr Biol..

[evv008-B6] Ellegren H (2007). Faced with inequality: chicken do not have a general dosage compensation of sex-linked genes. BMC Biol..

[evv008-B7] Ferreira A, Cella D, Mesa A, Virkki N (1984). Cytology and systematical position of Stylopids (Strepsiptera). Hereditas.

[evv008-B8] Gelbart M, Kuroda M (2009). *Drosophila* dosage compensation: a complex voyage to the X chromosome. Development.

[evv008-B9] Harrison PW, Mank JE, Wedell N (2012). Incomplete sex chromosome dosage compensation in the Indian meal moth, *Plodia interpunctella*, based on de novo transcriptome assembly. Genome Biol Evol..

[evv008-B10] Heard E, Disteche CM (2006). Dosage compensation in mammals: fine-tuning the expression of the X chromosome. Genes Dev..

[evv008-B11] Julien P (2012). Mechanisms and evolutionary patterns of mammalian and avian dosage compensation. PLoS Biol..

[evv008-B12] Langmead B, Salzberg SL (2012). Fast gapped-read alignment with Bowtie 2. Nat Methods..

[evv008-B13] Leder EH (2010). Female-biased expression on the X chromosome as a key step in sex chromosome evolution in threespine sticklebacks. Mol Biol Evol..

[evv008-B14] Li H, Durbin R (2009). Fast and accurate short read alignment with Burrows-Wheeler transform. Bioinformatics.

[evv008-B15] Li J (2013). Odoriferous Defensive stink gland transcriptome to identify novel genes necessary for quinone synthesis in the red flour beetle, *Tribolium castaneum*. PLoS Genet..

[evv008-B16] Li R (2009). SOAP2: an improved ultrafast tool for short read alignment. Bioinformatics.

[evv008-B17] Lin F, Xing K, Zhang J, He X (2012). Expression reduction in mammalian X chromosome evolution refutes Ohno’s hypothesis of dosage compensation. Proc Natl Acad Sci U S A..

[evv008-B18] Mank JE (2013). Sex chromosome dosage compensation: definitely not for everyone. Trends Genet..

[evv008-B19] Mank JE, Hosken DJ, Wedell N (2011). Some inconvenient truths about sex chromosome dosage compensation and the potential role of sexual conflict. Evolution.

[evv008-B20] Meyer B (2000). Sex in the worm: counting and compensating X-chromosome dose. Trends Genet..

[evv008-B21] Nguyen D, Disteche C (2006). Dosage compensation of the active X chromosome in mammals. Nat Genet..

[evv008-B22] Niehuis O (2012). Genomic and morphological evidence converge to resolve the enigma of Strepsiptera. Curr Biol..

[evv008-B23] Ohno S (1967). Sex chromosomes and sex linked genes.

[evv008-B24] Pessia E, Engelstadter J, Marais GA (2013). The evolution of X chromosome inactivation in mammals: the demise of Ohno’s hypothesis?. Cell Mol Life Sci..

[evv008-B25] Pessia E, Makino T, Bailly-Bechet M, McLysaght A, Marais GA (2012). Mammalian X chromosome inactivation evolved as a dosage-compensation mechanism for dosage-sensitive genes on the X chromosome. Proc Natl Acad Sci U S A..

[evv008-B26] Prince EG, Kirkland D, Demuth JP (2010). Hyperexpression of the X chromosome in both sexes results in extensive female bias of X-linked genes in the flour beetle. Genome Biol Evol..

[evv008-B27] Rastelli L, Kuroda MI (1998). An analysis of maleless and histone H4 acetylation in *Drosophila melanogaster* spermatogenesis. Mech Dev..

[evv008-B28] Roberts A, Pachter L (2013). Streaming fragment assignment for real-time analysis of sequencing experiments. Nat Methods..

[evv008-B29] Vicoso B, Bachtrog D (2009). Progress and prospects toward our understanding of the evolution of dosage compensation. Chromosome Res..

[evv008-B30] Vicoso B, Bachtrog D (2013). Reversal of an ancient sex chromosome to an autosome in *Drosophila*. Nature.

[evv008-B31] Vicoso B, Emerson JJ, Zektser Y, Mahajan S, Bachtrog D (2013). Comparative sex chromosome genomics in snakes: differentiation, evolutionary strata, and lack of global dosage compensation. PLoS Biol..

[evv008-B32] Wiegmann BM (2009). Single-copy nuclear genes resolve the phylogeny of the holometabolous insects. BMC Biol..

[evv008-B33] Zhou Q, Bachtrog D (2012). Sex-specific adaptation drives early sex chromosome evolution in *Drosophila*. Science.

