# Supplementary material for: Partial Dosage Compensation in Strepsiptera, a Sister Group of Beetles
Source: Genome Biol Evol. 2015 Jan 18;7(2):591–600. doi: 10.1093/gbe/evv008 (PMC4350179; doi:10.1093/gbe/evv008)
Supplement: Supplementary Data [file supp_7_2_591__index.html]

Partial Dosage Compensation in Strepsiptera, a Sister Group of Beetles — Supplementary Data 

# Partial Dosage Compensation in Strepsiptera, a Sister Group of Beetles

## Supplementary Data

files

**Files in this Data Supplement:**

- Supplementary Data - pdf file
- Supplementary Data - docx file
- Supplementary Data - docx file
