# Supplementary material for: Partial Dosage Compensation in Strepsiptera, a Sister Group of Beetles
Source: Genome Biol Evol. 2015 Jan 18;7(2):591–600. doi: 10.1093/gbe/evv008 (PMC4350179; doi:10.1093/gbe/evv008)
Supplement: Supplementary Data [file supp_evv008_SupplFig.pdf]

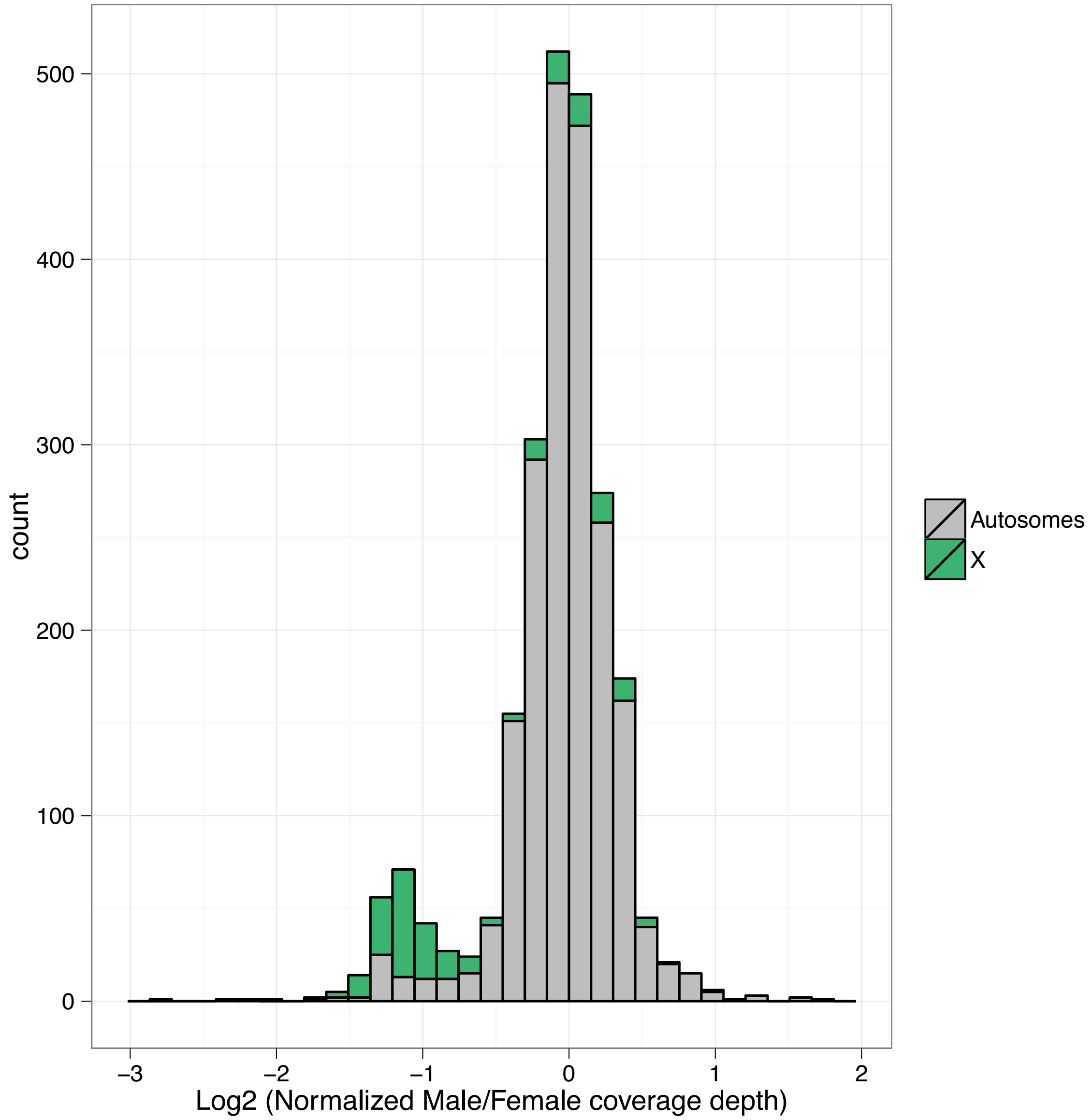

count
500
400
300
200
100
0
−3
−2
−1
0
1
2
Log2 (Normalized Male/Female coverage depth)
Autosomes
X

Figure S1

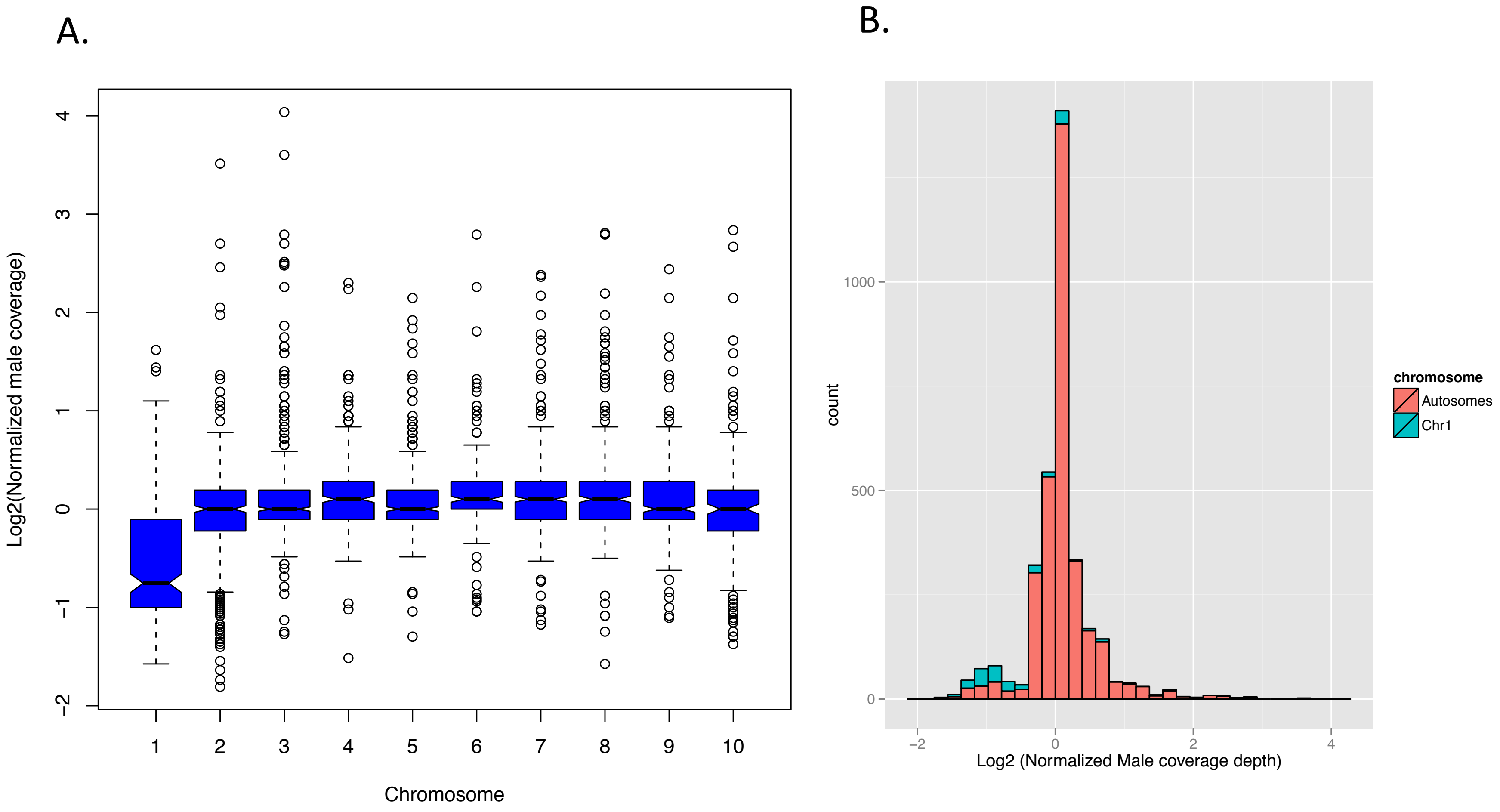
A.
Log2(Normalized male coverage)
4
3
2
1
0
−1
−2
1
2
3
4
5
6
7
8
9
10
Chromosome
B.
count
1000
500
0
−2
0
2
4
Log2 (Normalized Male coverage depth)
chromosome
Autosomes
Chr1

Figure S2

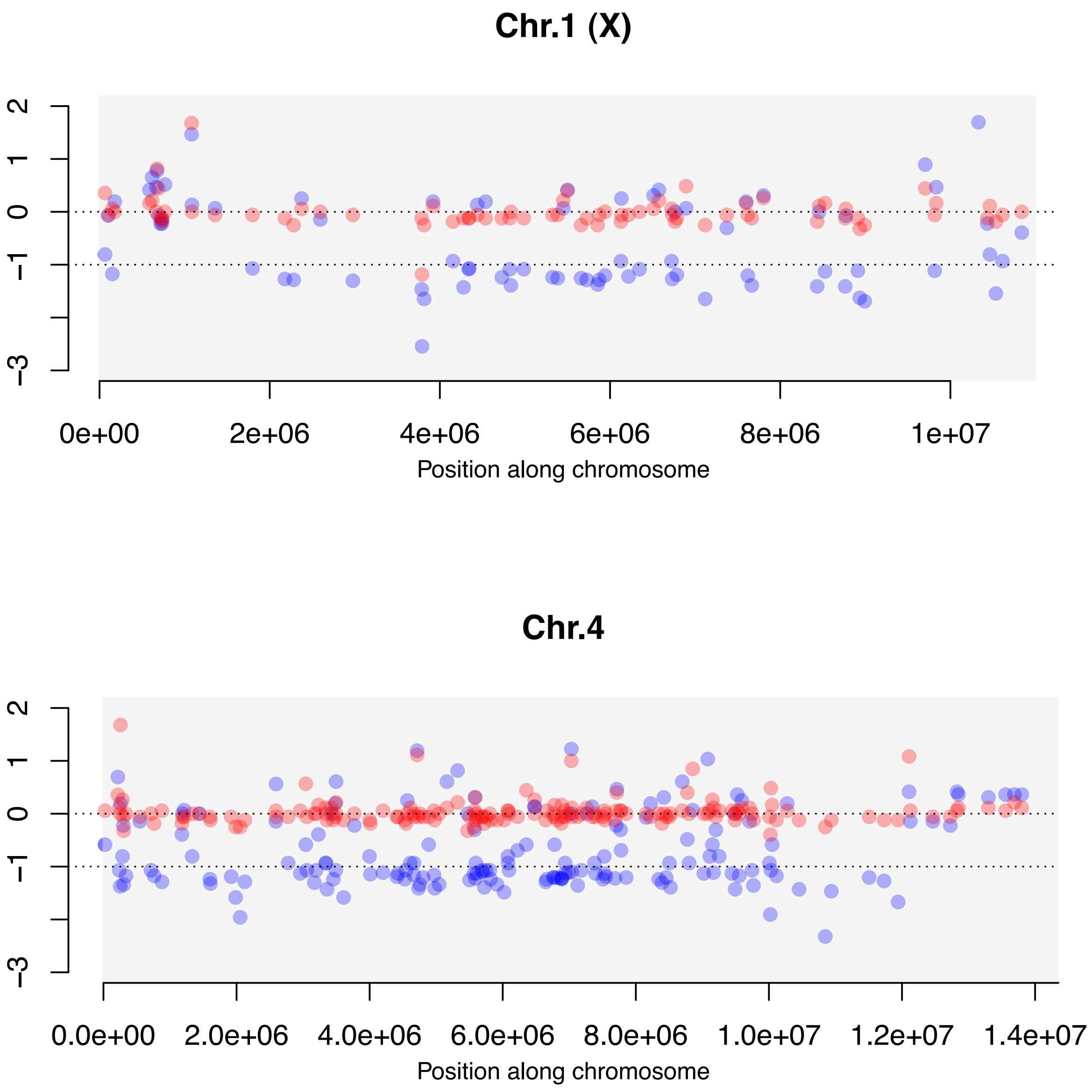

Chr.1 (X)
2
1
0
−1
−3
0e+00
2e+06
4e+06
6e+06
8e+06
1e+07
Position along chromosome
Chr.4
2
1
0
−1
−3
0.0e+00
2.0e+06
4.0e+06
6.0e+06
8.0e+06
1.0e+07
1.2e+07
1.4e+07
Position along chromosome

Figure S3

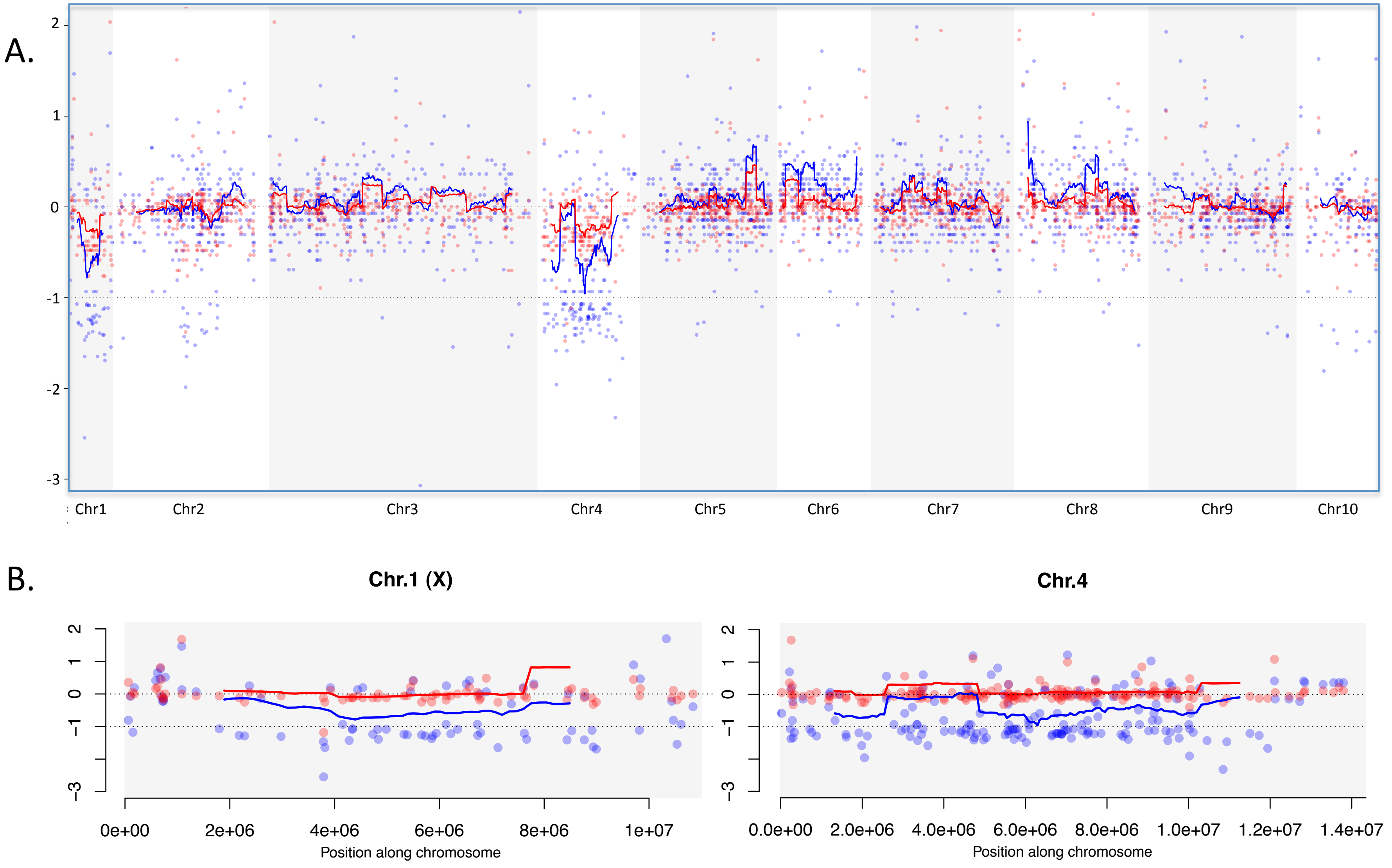
A.
2
1
0
-1
-2
-3
Chr1
Chr2
Chr3
Chr4
Chr5
Chr6
Chr7
Chr8
Chr9
Chr10
B.
Chr.1 (X)
Chr.4
2
1
0
−1
−3
0e+00
2e+06
4e+06
6e+06
8e+06
1e+07
Position along chromosome
0.0e+00
2.0e+06
4.0e+06
6.0e+06
8.0e+06
1.0e+07
1.2e+07
1.4e+07
Position along chromosome

Figure S4

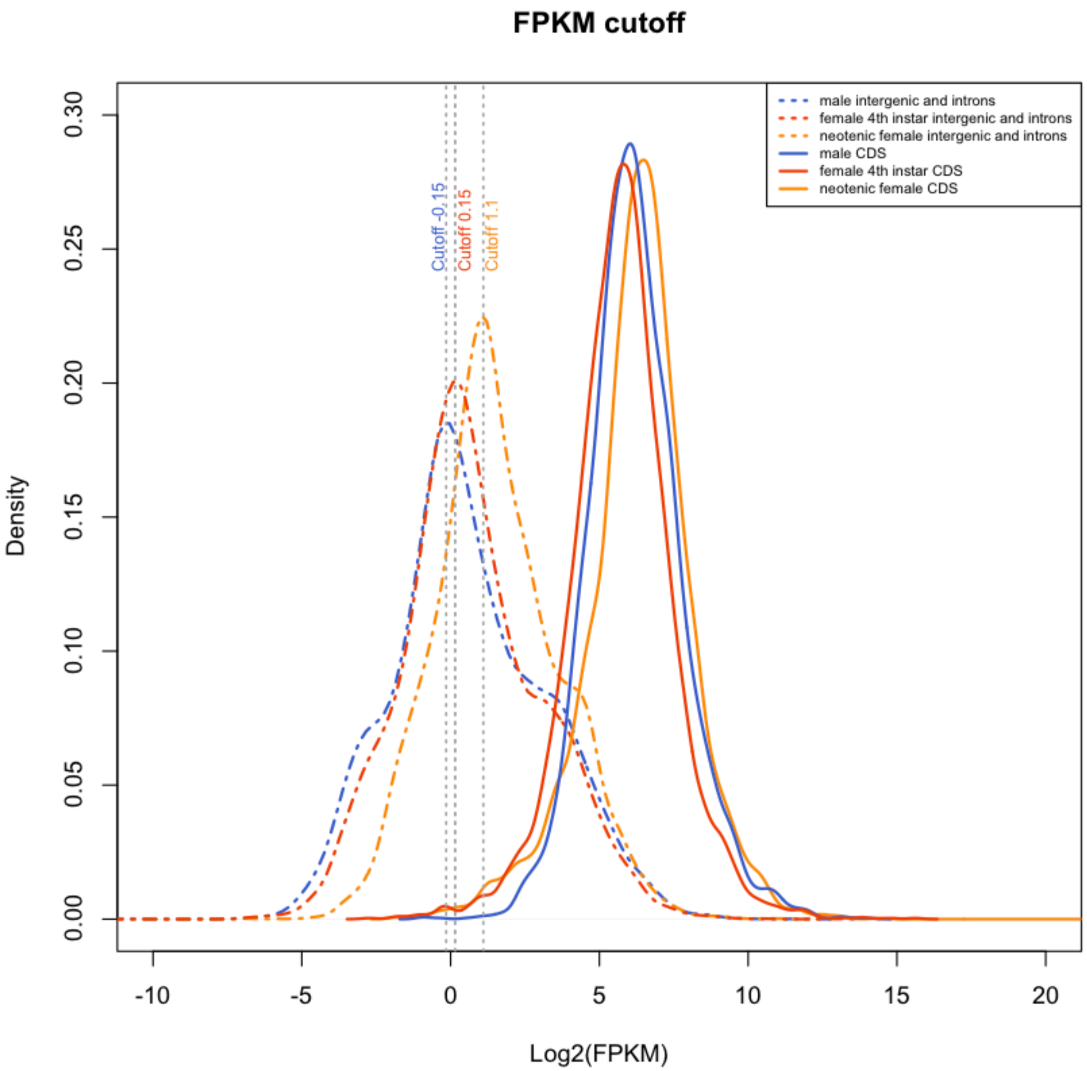
FPKM cutoff
male intergenic and introns
female 4th instar intergenic and introns
neotenic female intergenic and introns
male CDS
female 4th instar CDS
neotenic female CDS
Cutoff -0.15
Cutoff 0.15
Cutoff 1.1
Density
0.00
0.05
0.10
0.15
0.20
0.25
0.30
-10
-5
0
5
10
15
20
Log2(FPKM)

Figure S5

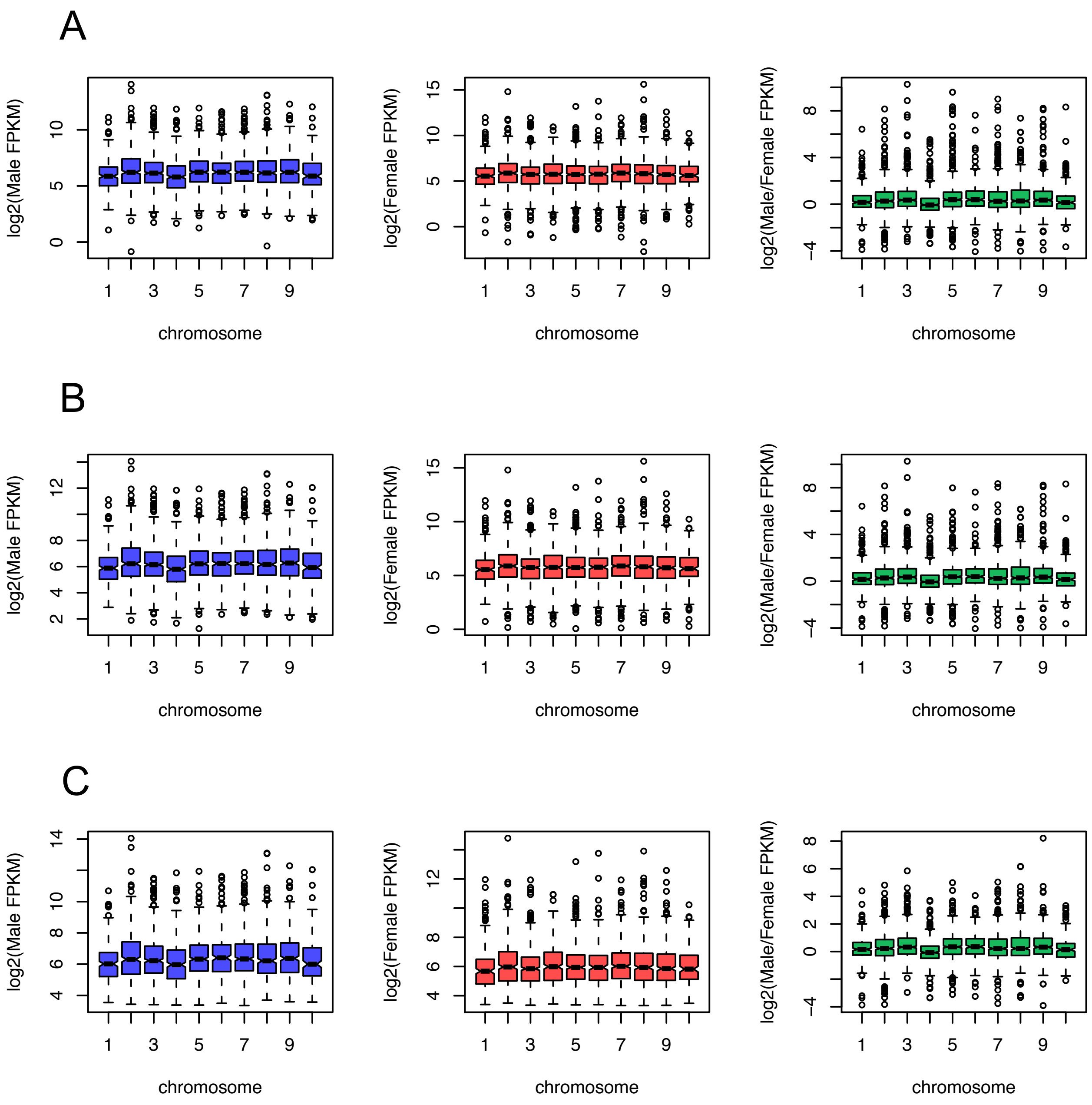
A
log2(Male FPKM)
chromosome
log2(Female FPKM)
chromosome
log2(Male/Female FPKM)
chromosome
B
log2(Male FPKM)
chromosome
log2(Female FPKM)
chromosome
log2(Male/Female FPKM)
chromosome
C
log2(Male FPKM)
chromosome
log2(Female FPKM)
chromosome
log2(Male/Female FPKM)
chromosome

Figure S6

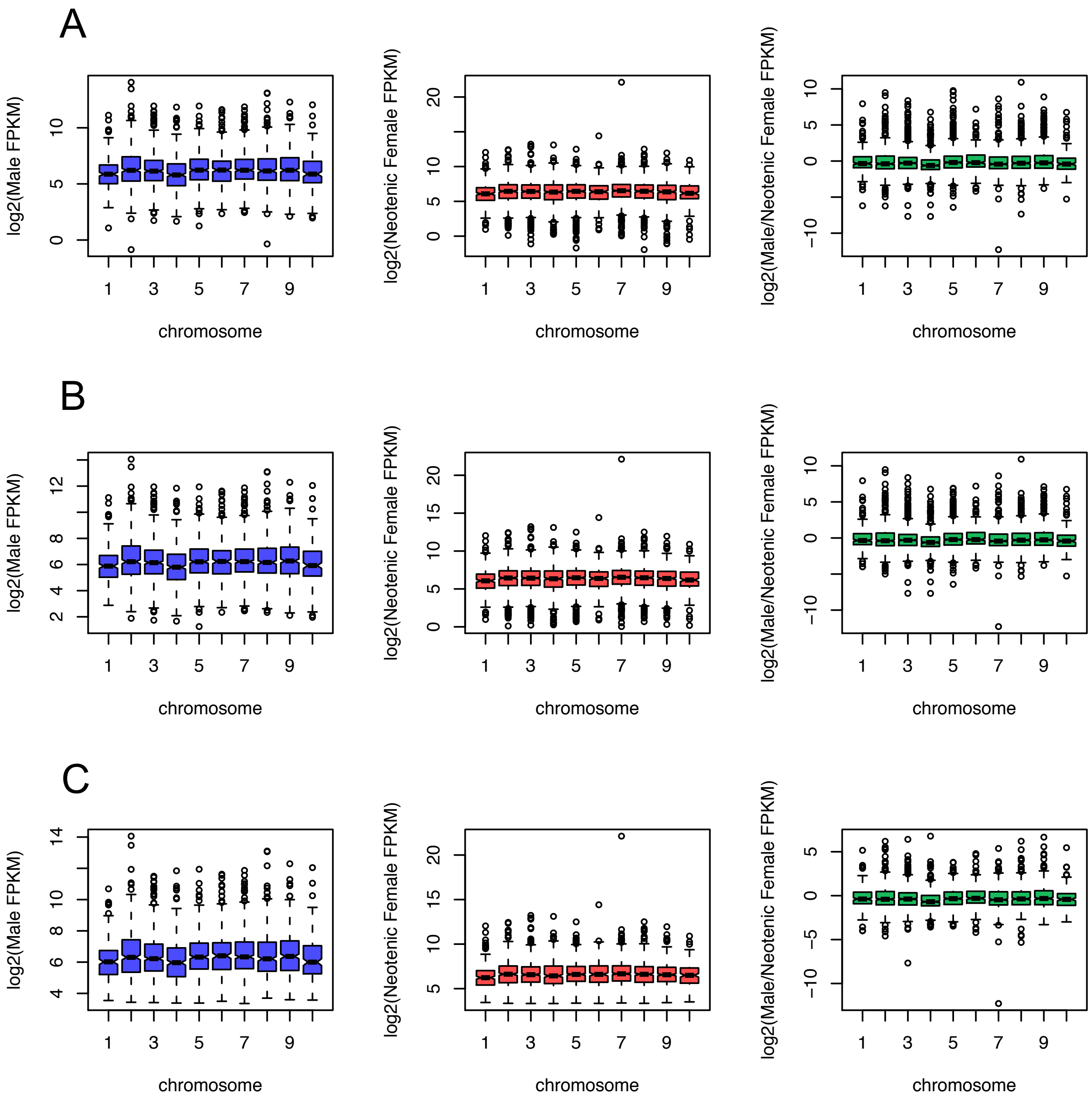

A
B
C
log2(Male FPKM)
log2(Neotenic Female FPKM)
log2(Male/Neotenic Female FPKM)
chromosome

Figure S7

A.

B.

C.

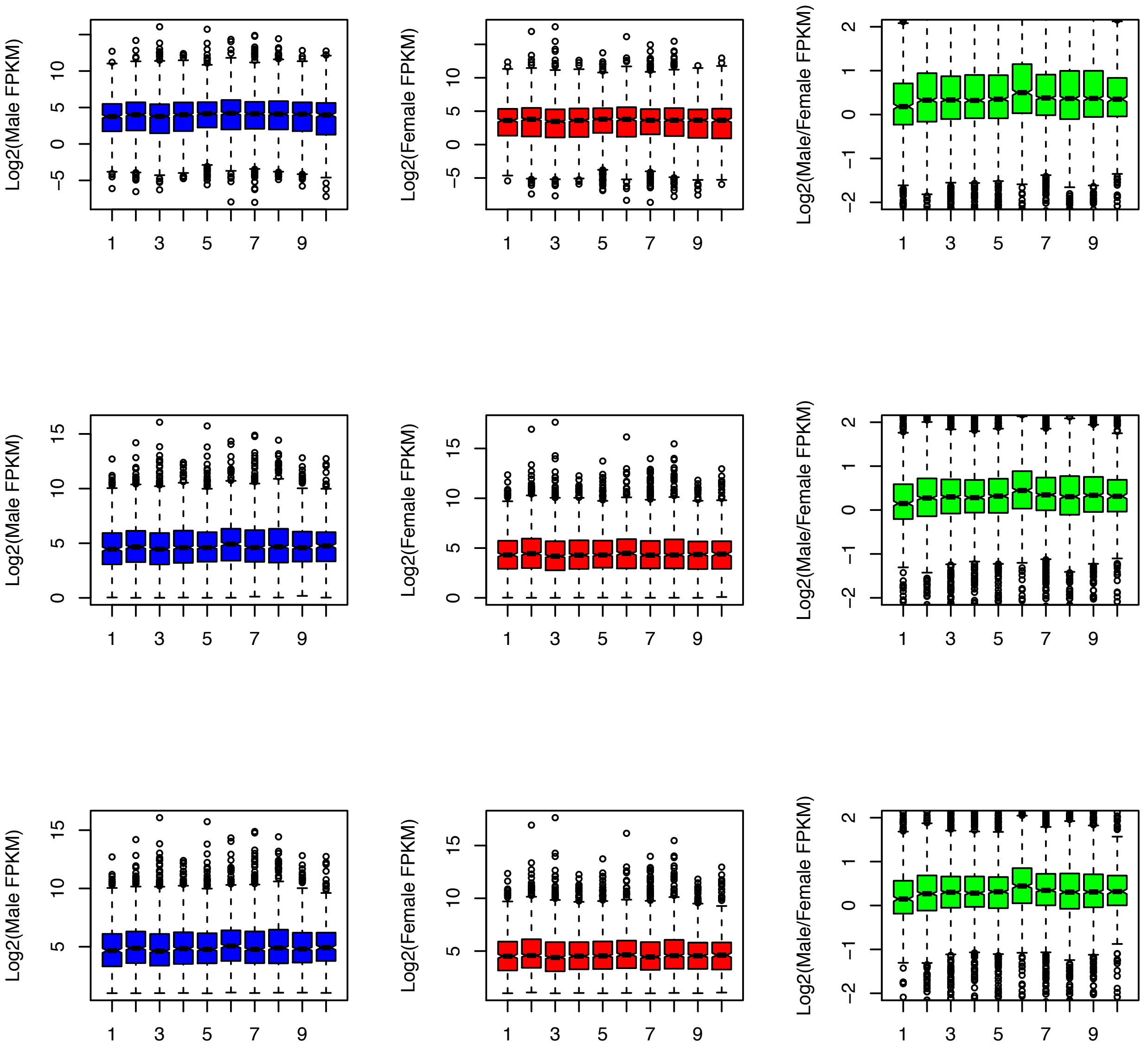

Figure S8

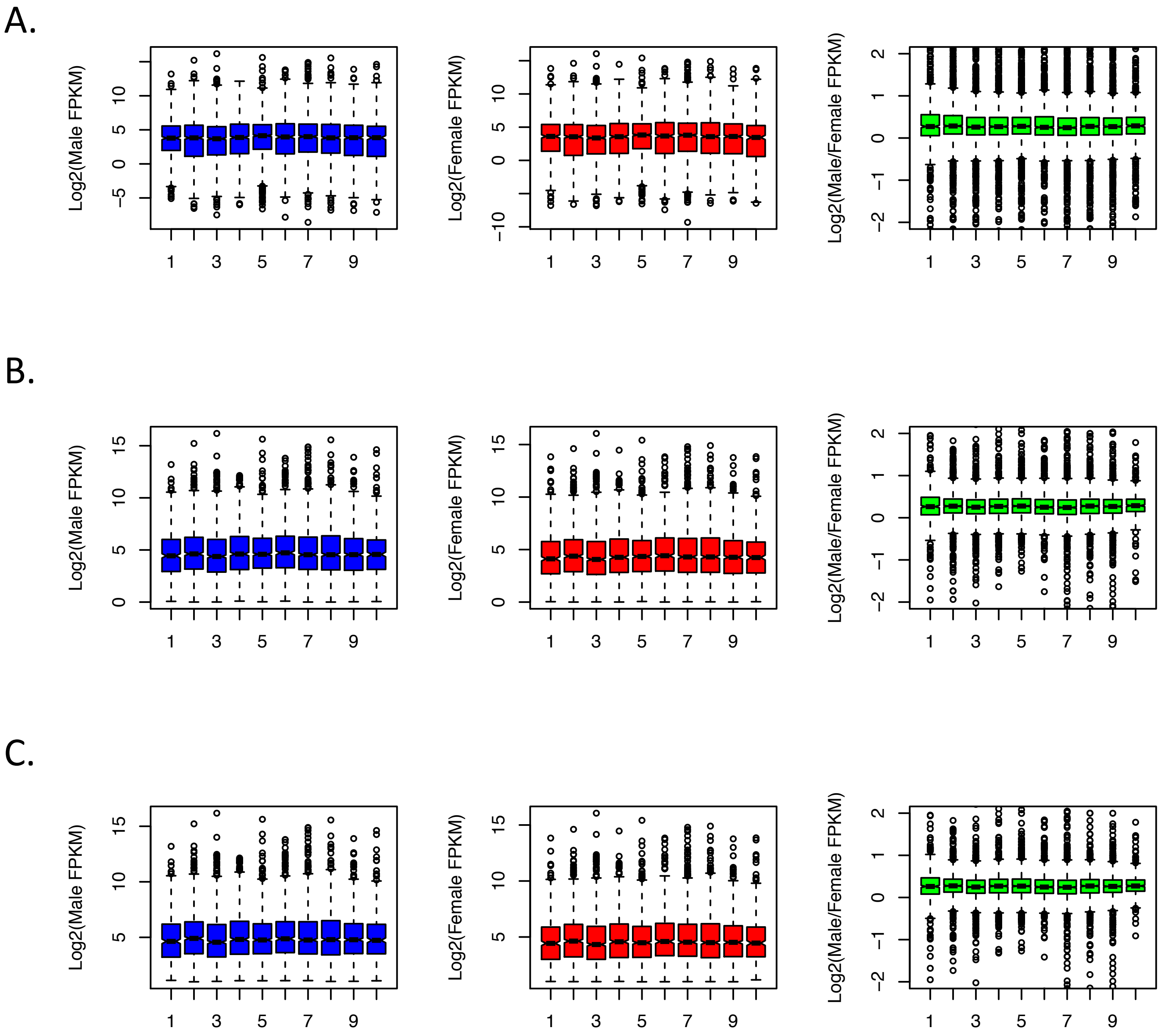
A.
B.
C.
Log2(Male FPKM)
Log2(Female FPKM)
Log2(Male/Female FPKM)
1
3
5
7
9

Figure S9

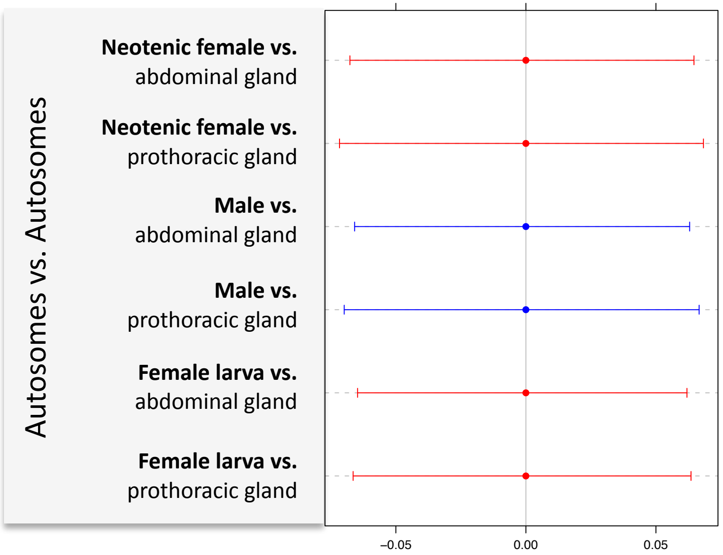

Autosomes vs. Autosomes
Neotenic female vs.
abdominal gland
Neotenic female vs.
prothoracic gland
Male vs.
abdominal gland
Male vs.
prothoracic gland
Female larva vs.
abdominal gland
Female larva vs.
prothoracic gland
−0.05
0.00
0.05

Figure S10

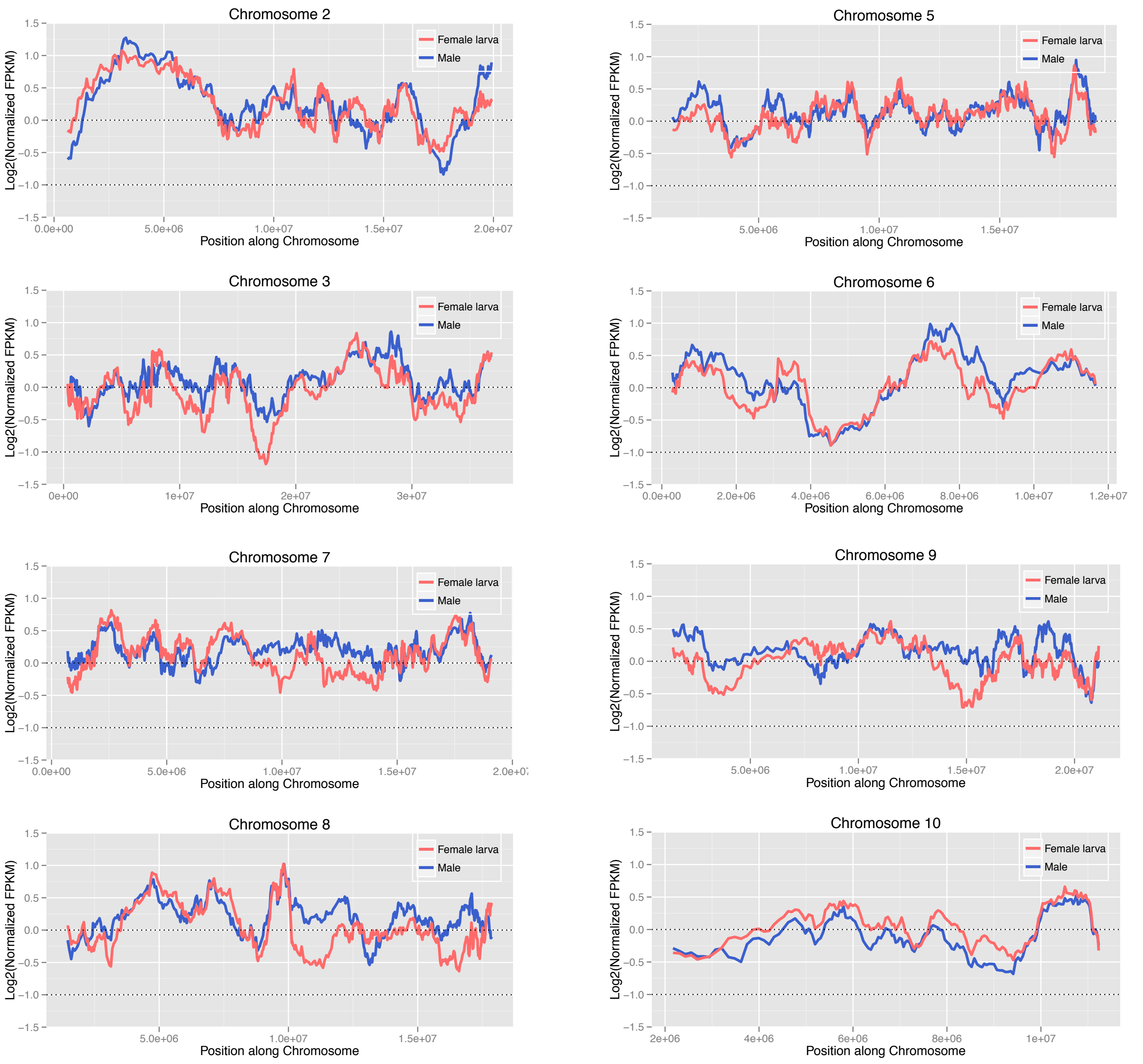
Chromosome 2
Chromosome 5
Chromosome 3
Chromosome 6
Chromosome 7
Chromosome 9
Chromosome 8
Chromosome 10
Female larva
Male
Log2(Normalized FPKM)
Position along Chromosome

Figure S11

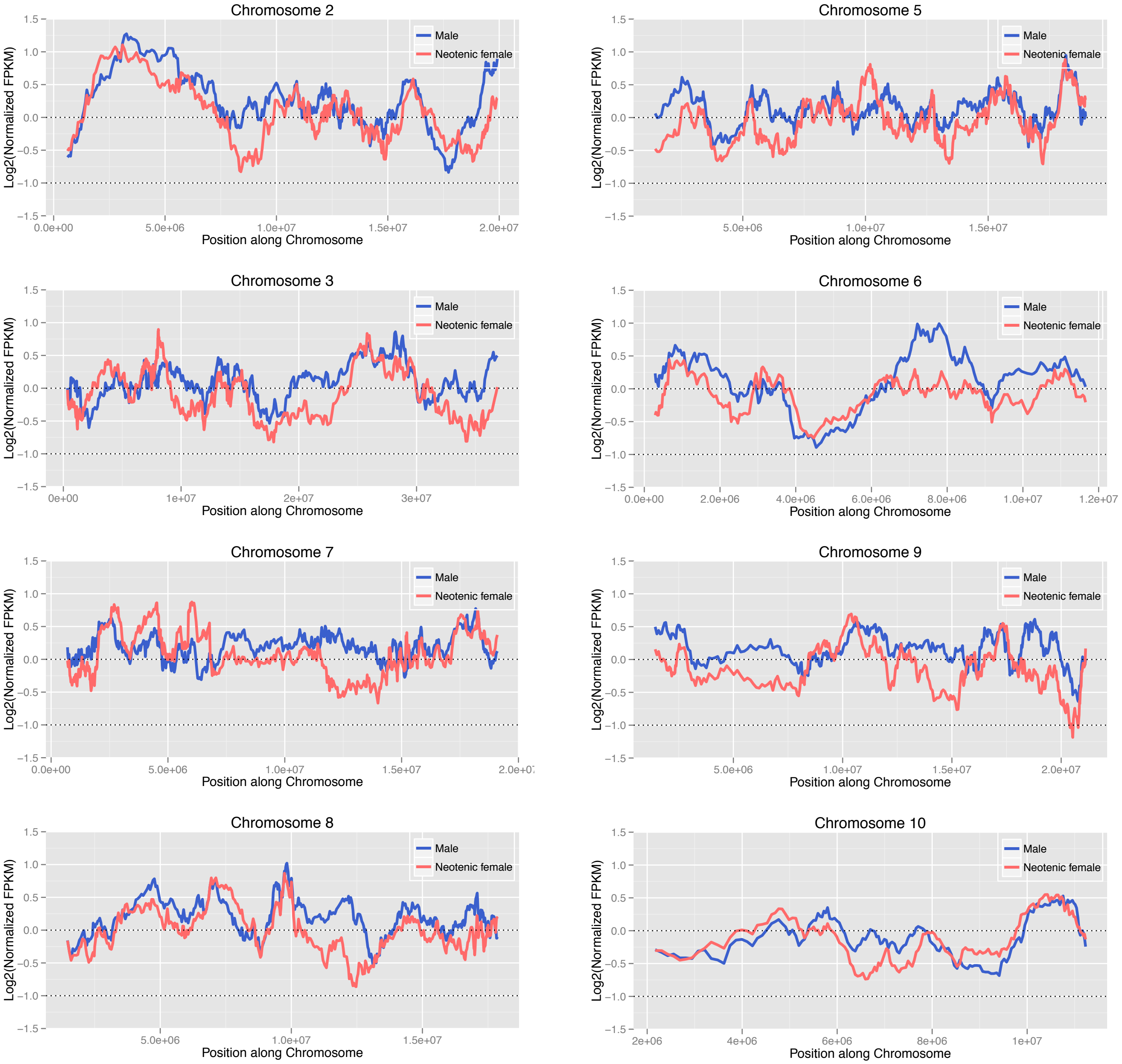
Chromosome 2
Chromosome 5
Chromosome 3
Chromosome 6
Chromosome 7
Chromosome 9
Chromosome 8
Chromosome 10
Male
Neotenic female
Log2(Normalized FPKM)
Position along Chromosome
1.5
1.0
0.5
0.0
−0.5
−1.0
−1.5
0.0e+00
5.0e+06
1.0e+07
1.5e+07
2.0e+07
0e+00
1e+07
2e+07
3e+07
2.0e+06
4.0e+06
6.0e+06
8.0e+06
1.2e+07
2e+06
4e+06
6e+06
8e+06
1e+07

Figure S12

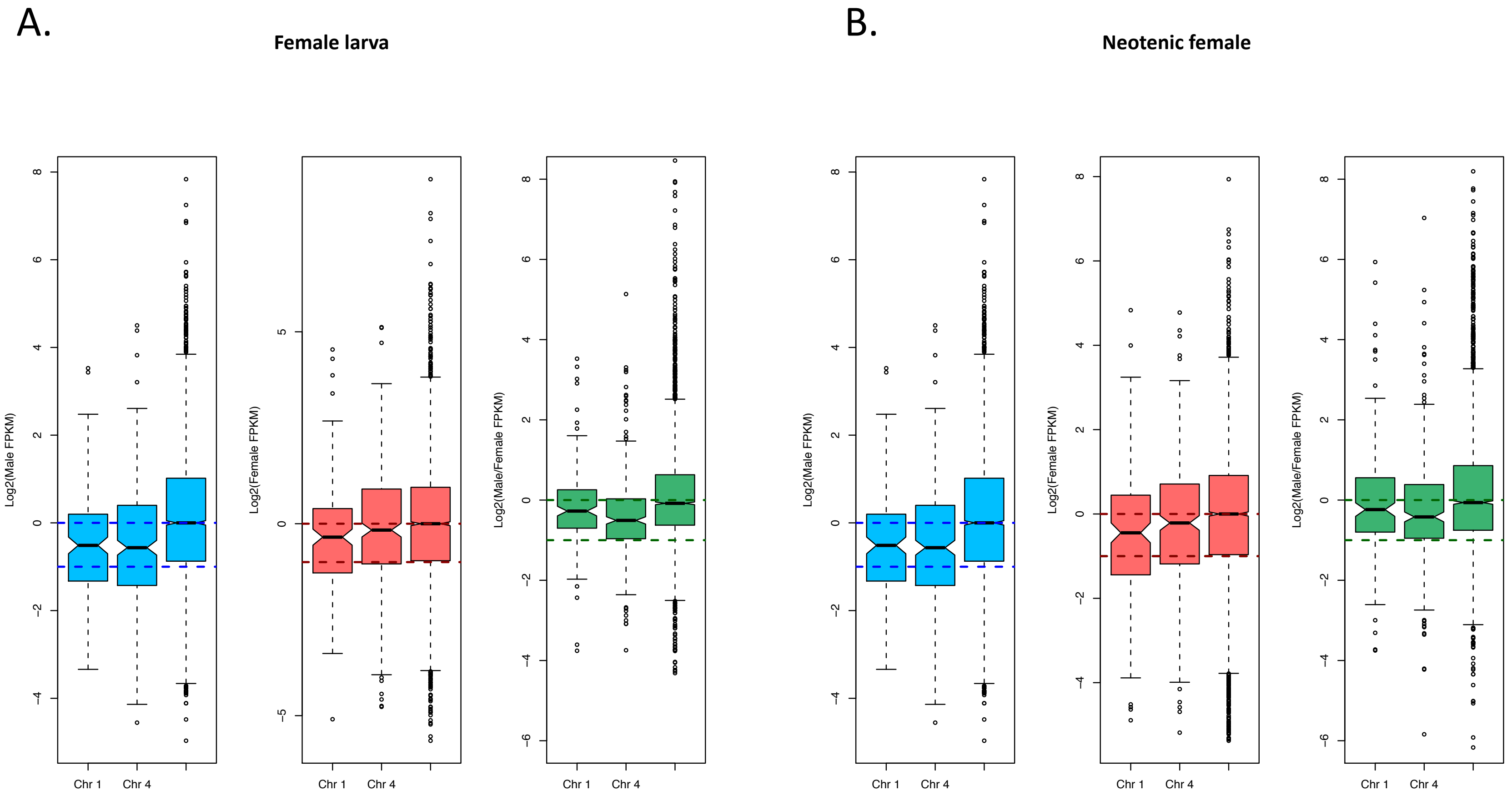

A.
Female larva
B.
Neotenic female
Log2(Male FPKM)
Log2(Female FPKM)
Log2(Male/Female FPKM)
Chr 1
Chr 4

Figure S13
