# Supplementary material for: Partial Dosage Compensation in Strepsiptera, a Sister Group of Beetles
Source: Genome Biol Evol. 2015 Jan 18;7(2):591–600. doi: 10.1093/gbe/evv008 (PMC4350179; doi:10.1093/gbe/evv008)
Supplement: Supplementary Data [file supp_evv008_New_Microsoft_Office_Word_Document.docx]

**Supplementary Figures**

**Supplementary** **Fig1**: Histogram of Log2 male/female coverage. Scaffolds that map to chromosomes 1 and 4 of *T. castaneum* are shown in green. The bimodal distribution in coverage suggests that a substantial fraction of the genome is sex-linked in *Xenos*, with the peak with reduced male/female coverage corresponding to scaffolds that are X-linked in *X. vesparum*.

**Supplementary Fig S2.** Coverage analysis in *M. moldrzyki.* (A) Boxplot of Log2 of normalized male coverage for scaffolds mapping to chromosomes 1-10 of *T*. *castaneum.* (B) Histogram of log2 of normalized male coverage. The bimodal distribution shows clear distinction between the coverage of the X-linked scaffolds and the autosomes. Scaffolds mapping to chromosome 1 in *Tribolium* are mostly X-linked and correspond to the lower peak.

**Supplementary** **Fig S3.** Log2 of the coverage densities of males (in blue) and females (in red) for scaffolds that mapped to chromosome 1 and 4 in *Tribolium*.

**Supplementary** **Fig S4.** Sliding window analysis of scaffolds mapped along the *Tribolium* genome. (A) Log2 of coverage densities of males (in blue) and females (in red) for the scaffolds that mapped to the ten chromosomes in *T. castaneum*. The lines represent a sliding window along the chromosomes, with a window size of 30 genes. Chromosomes 1 and 4 show a clear drop in coverage for males as compared to females. (B) Same as (A) but zoomed in into chromosome 1 and chromosome 4.

**Supplementary Fig S5**. Density plot of log(2) of the FPKM values for coding sequences (in continuous lines) and introns and intergenic regions (in dashed lines). Male is shown in blue, neotenic adult female is shown in orange and female larva is shown in red. The peaks for the introns and intergenic regions are used to determine the FPKM cutoff for the coding sequences for each sample.

**Supplementary Fig. S6**. Boxplot of Log2 of the expression in the 4th instar female larva (in red), male (in blue), and male/female (in green). Three different FPKM cutoffs were used, (A) 0 (B)1 and (C) 10 . In each case for the 4^th^ chromosome, the distribution was significantly different than the rest of the autosomes (p-values < 2.2e-16 , 2.824e-16 and < 2.2e-16 for FPKM cutoff of 0,1 and 10, respectively).

**Supplementary Fig. S7**. Boxplot of Log2 of the expression in neotenic adult female (in red), male (in blue), and male/female (in green). Three different FPKM cutoffs were used, (A) 0 (B) 1 and (C) 10. In each case for the 4^th^ chromosome, the distribution was significantly different than the rest of the autosomes (p-values 1.032e-06, 1.748e-06 and 1.007e-08 for FPKM cutoff of 0, 1 and 10, respectively).

**Supplementary Fig. S8.** Expression analysis of abdominal glands from *T*. *castaneum*

Log2 of FPKM values using cutoffs (A) FPKM>0 (B) FPKM>1 and (C) FPKM>2 for FPKM for chromosome 1-10. For all three cutoffs, no significant hypertranscription of the X is detected in females and all chromosomes are expressed at similar levels. Chromosome 1 in males is expressed at a slightly lower level for all three cutoffs (Wilcoxon test p-values 0.047, 0.055 and 0.01 for FPKM cutoff 0,1 and 2 respectively when comparing the expression of chromosome 1 with that of the autosomes; and Wilcoxon test p-values 1.222e-06, 2.461e-08, 2.769e-08 for FPKM cutoff 0,1 and 2 respectively when comparing male/female expression of chromosome 1 versus the male/female expression of the autosomes.)

**Supplementary Fig S9**. Expression analysis of prothoracic glands from *T. castaneum*

Log2 of FPKM values using cutoffs (A) FPKM>0 (B) FPKM>1 and (C) FPKM>2. For both male and female, for all three FPKM cutoffs, all chromosomes are found to be expressing at similar levels. No reduction in expression is detected for chromosome 1 in males, unlike what was found for the abdominal glands (Fig. S8).

**Supplementary Fig. S10.** Ancestral expression analysis of Xenos sex chromosomes, using expression values in male and female *T. castaneum* as a proxy for ancestral expression values. Expression of autosomes in *X. vesparum* relative to the expression of autosomes in *T*. *castaneum*. Female *X.* *vesparum* are shown in red and male *X. vesparum* is shown in blue. Expression of each *X. vesparum* sample is compared to the expression in both abdominal (top) and prothoracic glands (bottom) of *Tribolium* for each sex separately.

**Supplementary Fig. S11. Sliding window analysis of gene expression in autosomes**. Log2 of normalized FPKM values of *Xenos* males and females mapped along chromosomes 2,3,5,6,7,8,9,10 of *T. castaneum*. Male is shown in blue and female larva is shown in red.

**Supplementary Fig. S12. Sliding window analysis of gene expression in autosomes**. Log2 of normalized FPKM values of *Xenos* males and females mapped along chromosomes 2,3,5,6,7,8,9,10 of *T. castaneum*. Male is shown in blue and neotenic adult female is shown in red.

**Supplementary Fig. S13. Dosage compensation analysis** for scaffolds assigned as X-linked based on coverage. (A, B) Boxplot of Log2 of expression in (A) 4^rd^ instar female larva and (B) neotenic adult female (in red), male (in blue), and male/female (in green). The distribution of log2(Male/Female) FPKM values for chromosome 4 is significantly different than that of the autosomes with a Wilcoxon test (p-value of 6.5e-16 for 4^th^ instar female larva; p-value of 3.1e-06 for neotenic adult female).
