# Supplementary material for: Partial Dosage Compensation in Strepsiptera, a Sister Group of Beetles
Source: Genome Biol Evol. 2015 Jan 18;7(2):591–600. doi: 10.1093/gbe/evv008 (PMC4350179; doi:10.1093/gbe/evv008)
Supplement: Supplementary Data [file supp_evv008_SupplTables.docx]

| Table S1. Assembly statistics for the *Xenos vesparum* genome | |
| --- | --- |
| Scaffold N50 | 12,491 bp |
| Total Number of Scaffolds | 11,895 |
| Scaffolds above 1Kb | 11,243 |
| Longest scaffold length | 212,630 bp |
| Average scaffold length | 6,842 bp |
| Length of assembled genome | 81,397,174 bp |
| Scaffolds that mapped to *Tribolium* | 2,291 |

| Table S2. Assembly statistics for the *Xenos vesparum* transcriptome | |
| --- | --- |
| Contig N50 | 584bp |
| Scaffold N50 | 1,196 bp |
| Total number of scaffolds | 23,344 |
| Scaffolds above 300bp | 11,509 |
| Longest scaffold length | 10,874 bp |
| Average scaffold length | 614 bp |
| Length of assembled transcriptome | 13,802,567 bp |
| Number of genes obtained after filtering and mapping to *Tribolium* | 4,413 |
